# Infection with Opportunistic Bacteria Triggers Severe Pulmonary Inflammation in Lupus-Prone Mice

**DOI:** 10.1155/2019/1701367

**Published:** 2019-09-03

**Authors:** Wenchao Li, Weiwei Chen, Saisai Huang, Xiaojun Tang, Genhong Yao, Lingyun Sun

**Affiliations:** The Affiliated Drum Tower Hospital of Nanjing University Medical School, Nanjing, Jiangsu 210008, China

## Abstract

Infection is a common cause of hospitalization and mortality in patients with systemic lupus erythematosus (SLE). How the underlying immune dysfunctions affect the antimicrobial immunity remains largely unknown. In the present study, employing the pulmonary infection model, we determined the antimicrobial defence of lupus-prone mice. After infecting with opportunistic bacterium *Haemophilus influenzae* (Hi), lupus-prone mice (B6/lpr) exhibited inefficient bacterial elimination and recovered slowly. They generated severer inflammation at the early stage of infection, as excessive accumulation of neutrophils and enhanced production of proinflammatory cytokines were observed in the lung. In addition, a large number of apoptotic cells were detected in the lungs of B6/lpr mice. For adaptive immune responses, B6/lpr mice were capable to generate enough protective Hi-specific Th17 cells. They evoked stronger Hi-specific *γδ* T17 response in both lungs and spleens. Unexpectedly, both CD4 and *γδ* T cells from lupus-prone mice showed deficiency in IFN-*γ* production. For humoral immune responses, compared with those of WT mice, the concentrations of Hi-specific IgA, IgM, and IgG, especially IgG, were significantly higher in the B6/lpr mice. Our findings suggest that lupus mice are capable to generate antibacterial immune responses; however, the overwhelming inflammation and overactivated immune responses increase the severity of infection.

## 1. Introduction

Systemic lupus erythematosus (SLE) is a complicated autoimmune disease with ill-elucidated etiology. Aside from the active disease, infection is responsible for approximately 30% of all deaths and represents a leading cause of mortality in SLE [1]. In addition to common infections, SLE patients are frequently infected by opportunistic pathogens, exhibiting a weakened immune defence [2]. Although long-term exposure to immunosuppressive agents seriously impairs the antimicrobial immunity, underlying immune dysregulation of SLE patients also contributes to the increased incidence of infection.

According to previous studies, inherent defects in both innate and acquired immune responses predispose SLE patients to infection. Macrophages and neutrophils are the first-line defence against invading pathogen. However, impaired phagocytosis by macrophages and neutrophils has been reported in SLE patients [3]. In addition, clinical data demonstrate that cochemotaxin inhibitor in lupus serum strongly inhibits the chemotaxis of neutrophils by C5a des Arg [4]. Cytotoxic T cells are important for controlling intracellular bacterial infection. Compared with healthy controls, SLE patients have significantly fewer SLAMF4 CD8 T cells. A reduced expression of SLAMF4 leads to the impaired cytotoxic function of CD8 T cells [5]. When infected by *T. gondii*, inhibited antigen-specific IFN-*γ* response prevents lupus-prone mice from clearing infection effectively and is likely responsible for the increased mortality [6]. Except for the aforementioned cellular defects, during the progress of disease, autoantibodies against key components required for antimicrobial responses could be generated and cause recurrent infection [7]. Nevertheless, the mentioned information is far more enough, and many problems remain unsolved.

To investigate the increased susceptibility, we infected C57BL/6MRL/Fas*^lpr^* (B6/lpr) mice with *Haemophilus influenzae* (Hi), a common cause of respiratory infection frequently isolated from SLE patients [8], and examined both the cellular and humoral immune responses induced by infection.

## 2. Materials and Methods

### 2.1. Animals

Female C57BL/6 (B6) and C57BL/6MRL/Fas*^lpr^* (B6/lpr) mice (22 weeks old; Nanjing Medical University Animal Core) were used in all experiments. Animals were maintained in the animal facility at Nanjing Drum Tower Hospital. All experimental protocols were approved by the Ethics Committee for Animal Research in the Affiliated Drum Tower Hospital (No. 20160802).

### 2.2. Preparation of Bacteria

*H. influenzae* (strain 86-028NP) glycerol stocks were stripped onto brain heart infusion agar plates supplemented with nicotinamide adenine dinucleotide (5 *μ*g/ml) and hemin (10 *μ*g/ml) (sBHI). Bacteria were grown at 37°C in a CO_2_ incubator overnight. Then, Hi colonies were inoculated into 5 ml of fresh sBHI broth and subjected to shaking to reach OD_600_ = 0.45. Bacteria were subsequently harvested, washed, and resuspended in sterile PBS at a concentration of 3 × 10^9^ CFU/ml. For preparation of the heat-killed Hi, the resulting bacterial suspension was heated to 65°C for 30 min, and 50 *μ*l of the heated bacteria was plated to ensure 100% bacterial killing.

### 2.3. Pulmonary Infection Model

Mice were anaesthetized by intraperitoneal injection of 100 *μ*l ketamine/xylazine (150 mg/10 mg/kg) and inoculated with 1 × 10^8^ CFU of Hi in 30 *μ*l PBS intranasally (Day 0). To monitor the bodyweight change, mice were weighed daily until fully recovered (Day 10). To enumerate bacteria in the lung, mice were sacrificed on Day 2 and Day 10 after infection. Bronchial alveolar lavage fluid (BALF) and lungs were collected. The left lobes of the lungs were homogenized in 1 ml sterile PBS. Tenfold serial dilutions of the homogenates and BALF were prepared in PBS and plated on sBHI plates. Colonies were counted after overnight culture at 37°C.

### 2.4. Bronchial Alveolar Lavage

BALF was obtained by cannulating the trachea with a 20-gauge catheter. The lungs were lavaged with cold PBS twice (each aliquot 0.8 ml); total returns averaged 1-1.4 ml. BALF was then centrifuged at 400g for 5 min at 4°C. The cell-free supernatants were stored at -80°C for later analysis. The cell pellet was resuspended in 0.5 ml PBS. An aliquot of the cell suspension was processed immediately for total cell counts with a hemocytometer.

### 2.5. Isolation of Lung and Spleen Cells

Lungs were minced and incubated at 37°C in Iscove's DMEM containing 3.5 mg/ml collagenase A (Roche) and 2.5 mg/ml DNase I (Sigma), then mashed through a 40 *μ*m cell strainer (BD Falcon). The spleen was excised into small pieces and placed onto a cell strainer attached to a 50 ml conical tube. Then, fragments were pressed through the strainer using the plunger end of a 5 ml syringe. Cell suspension was centrifuged, and red cells were removed by adding lysing solution (BD Pharmingen).

### 2.6. Measurement of BALF Protein and Cytokine/Chemokines

The total protein concentration in BALF was measured by the bicinchoninic acid method (BCA assay; Jiancheng, Nanjing, Jiangsu, China). According to manufacturer's instructions, BALF levels of cytokines were measured with ELISA kits (R&D Systems) for murine IL-6, IL-17, TNF-*α*, KC (CXCL-1), and MCP-1. Results were expressed in pg/ml. The detecting limitation of IL-6, IL-17, TNF-*α*, and KC (CXCL-1) is 7.5 pg/ml, while that of MCP-1 is 30 pg/ml.

### 2.7. Lung Pathology

The lungs were collected and fixed in 10% formalin. Paraffin-embedded lungs were cut into 5 *μ*m thick sections and subsequently stained with hematoxylin and eosin (H&E) for histological analysis. Following the reported grading guidelines [9], a pathologist blindly scored each lung injury using the following five categories listed in Table 1. Five fields per section from each sample were determined. The injury score was calculated as follows:

(1) $$\begin{array}{c} \text{Score}=\frac{\left(20\times A\right)+\left(14\times B\right)+\left(7\times C\right)+\left(7\times D\right)+\left(2\times E\right)}{\text{number of fields}\times 100}. \end{array}$$

### 2.8. Apoptosis Assay of the Lung

Apoptosis of lung cells was assessed by terminal deoxynucleotidyl transferase biotin-dUTP nick end labeling (TUNEL) staining with an *in situ* cell death detection kit (Roche, Germany). Ten fields per section from regions with cell apoptosis were examined at ×400 magnification. According to the percentage of TUNEL-positive cells, a five-point, semiquantitative, severity-based scoring system was used to assess the degree of apoptosis: 0 = normal lung parenchyma, 1 = 1–25%, 2 = 26–50%, 3 = 51–75%, and 4 = 76–100% of examined tissue [10].

### 2.9. Flow Cytometry

BALF and lung cells were stained at 4°C in RPMI 1640 medium containing 1% FBS after Fc*γ*RII/III blocking with anti-mouse CD16/CD32 (clone 93; eBioscience). Surface staining was performed with antibodies purchased from eBioscience (anti-CD45; clone 30-F11; anti-Ly6G (Gr-1), clone RB6-8C5; anti-CD11c, clone N418; and anti-CD11b, clone M1/70). Alveolar macrophages (CD11b^−^CD11c^+^) and neutrophils (CD11b^+^Gr-1^+^) were analysed.

For intracellular staining, lung and spleen cells were stimulated with heat-killed bacteria at MOI (multiplicity of infection) 100 for 16 h at 37°C with Golgi block added on the last 5 hours. Cells were then surface-stained with anti-CD4 (clone RM4-5; eBioscience) and anti-*γδ*TCR (clone GL-3; eBioscience) and followed by permeabilization with Cytofix-Cytoperm solution (BD Pharmingen). Then, the cells were stained with anti-IL-17A (clone 17B7; eBioscience) and anti-IFN-*γ* (clone XMG1.2; eBioscience). All samples were analysed with FACSCalibur. Data were analysed with FlowJo software.

### 2.10. Antibody Measurement by ELISA

Diluted heat-killed Hi (approximately 3 × 10^8^ CFU) was coated onto 96-well plates by incubation at 4°C overnight. Wells were blocked with 1% BSA in PBS, and twofold serial dilutions of BALF or sera were applied to the wells in duplicate at appropriate dilutions. Plates were incubated at 37°C for 1 hour. Antibodies were detected with goat anti-mouse IgG-HRP (H+L), IgA, and IgM (Proteintech). For detection, the TMB peroxidase substrate reagent set (BioLegend) was used according to the manufacturer's instructions. Optical densities were read at 450 nm with the microplate reader.

### 2.11. Statistical Analysis

All analyses were performed by using GraphPad. CFU data that fell below the limit of detection were assigned a value below that limit and were analysed by the unpaired *t*-test. The Kruskal-Wallis test was used to evaluate variance among all groups. If a significant variance was found, the Mann–Whitney test was used to determine significant differences between individual groups. *p* < 0.05 was considered to represent a statistically significant difference.

## 3. Results

### 3.1. Lupus-Prone Mice Exhibited Delayed Bacterial Clearance and Severer Lung Injury after Infection

**Figure 1:**
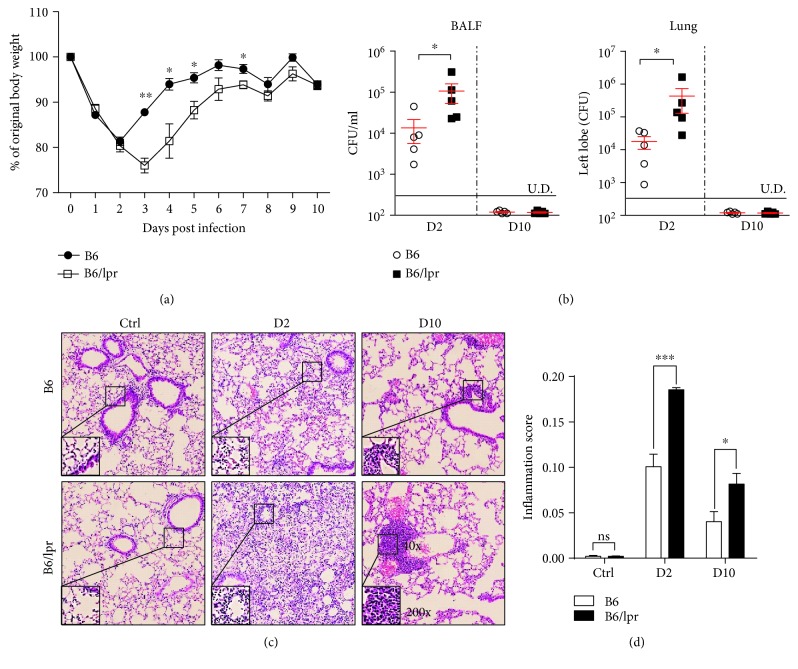
Lupus-prone mice exhibited delayed bacterial clearance and severer lung injury after infection. (a) B6 and B6/lpr mice were infected with 1 × 10^8^ CFU*H. influenzae* (strain 86-028NP) intranasally. Their bodyweight changes were monitored until fully recovered. B6 mice: circle; B6/lpr mice: square. Mice were infected as described before and sacrificed on Days 2 (D2) and 10 (D10) after infection. (b) Bacteria in the BALF and lungs were recovered and determined. Lung pathology was examined by H&E staining. (c) H&E staining of representative animals. Original magnification: 40x; inlets: 200x. (d) Histopathological mean inflammation scores of lungs. Data were expressed as means ± SEM. *n* = 3‐5 mice for each treatment group. ^∗^*p* < 0.05, ^∗∗^*p* < 0.01, and ^∗∗∗^*p* < 0.001. ns: no significant difference; U.D.: under the limit of detection. This experiment is representative of three individual experiments.

To investigate whether the inherent immune dysfunctions predispose lupus-prone mice to infections, B6/lpr mice, which have a spontaneous *FAS* mutation and recapitulate many features of lupus, were infected with *H. influenzae* (Hi, strain 86-028NP) intranasally. Then, bodyweight changes of B6/lpr mice were monitored and compared with those of B6 mice. We found that on Day 3 after infection, when B6 mice began to regain bodyweight, B6/lpr mice kept losing five percent of weight. In addition, they took more time to recover (Figure 1(a)).

Next, we determined the ability of B6/lpr mice to clear the invaded bacteria. On Day 2 and Day 10 after infection, Hi in the BALF and lungs were recovered and counted. Our data showed that B6/lpr mice could finally eliminate the invading Hi, as no bacteria were detected 10 days later. However, B6/lpr mice bore approximately 10-fold more bacteria than B6 mice at the early stage of infection (Day 2) (Figure 1(b)). Then, we examined the lung pathology by H&E staining. As the data show, few inflammatory cells were detected in the lungs of uninfected control B6 and B6/lpr mice (Figure 1(c)). However, on Day 2 after Hi infection, severer tissue damage and more infiltrating cells were observed in the lungs of B6/lpr mice. Moreover, on Day 10 after infection, when inflammation was resolved in B6 controls, there were still many inflammatory cells surrounding the bronchi of B6/lpr mice (Figures 1(c) and 1(d)).

**Figure 2:**
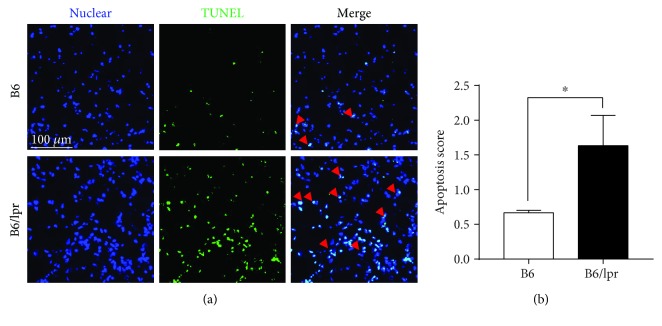
Infection induced elevated apoptosis of lung cells in the lupus-prone mice. B6 and B6/lpr mice were infected with Hi and sacrificed two days later. (a) Apoptosis of lung cells was determined by the TUNEL assay. The nuclei were counterstained with DAPI dye (blue) and TUNEL-positive nuclei (green fluorescence, indicated by red arrowheads in the merged photos). Scale bar = 100 *μ*m. (b) Apoptosis score for the lung was calculated. Data were expressed as means ± SEM. *n* = 3 mice for each treatment group; ^∗^*p* < 0.05. This experiment is representative of three individual experiments.

Since cell apoptosis is closely associated with tissue damage and inflammatory responses [11], we next determined the apoptosis of lung cells. On Day 2 after infection, the lungs of B6 and B6/lpr mice were collected and subjected to the TUNEL assay. Only a few cells underwent apoptosis in the B6 mice, while elevated apoptosis was detected in the B6/lpr mice (Figure 2). Together, these findings indicated that infection triggered exacerbated inflammatory responses in the lungs of lupus-prone mice.

### 3.2. Infection Induced Heightened Production of Inflammatory Cytokines in Lupus-Prone Mice

**Figure 3:**
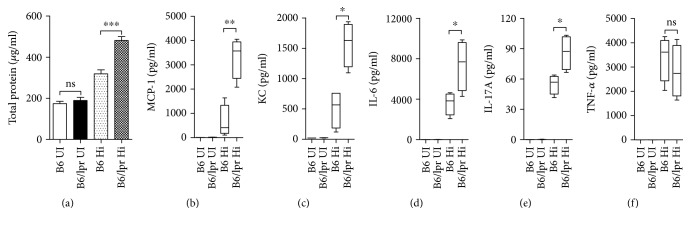
Infection induced heightened production of inflammatory cytokines in lupus-prone mice. B6 and B6/lpr mice were infected with Hi (B6 Hi and B6/lpr Hi). Uninfected mice were set as controls (B6 UI and B6/lpr UI). Two days after infection, all the mice were sacrificed and BALF was collected. (a) Protein levels were determined with the BCA assay. (b, c) Chemokines and (d–f) inflammation-related cytokines in the BALF were measured by ELISA. Data were expressed as means ± SEM. *n* = 3‐4 mice for each treatment group. ^∗^*p* < 0.05, ^∗∗^*p* < 0.01, and ^∗∗∗^*p* < 0.001. ns: no significant difference. This experiment is representative of three individual experiments.

On Day 2 after infection, concentrations of total protein in the BALF were assayed to assess pulmonary vascular leakage as a marker of acute lung injury. Hi induced a modest degree of lung injury in B6 mice, while increased vascular permeability was observed in the B6/lpr mice, as a higher protein level was detected (Figure 3(a)). Since chemokines and proinflammatory cytokines are important mediators of inflammatory responses, we next determined their productions in the lung. In BALF collected from uninfected B6 or B6/lpr mice, as expected, no production of such inflammatory-associated cytokines was detected. On Day 2 after infection, monocyte chemoattractant MCP-1 and neutrophil attractant KC increased dramatically; however, in the B6/lpr mice, their concentrations were much higher (503.5 ± 155.9 vs. 1571 ± 183; 638.5 ± 340.3 vs. 3320 ± 428.4) (Figures 3(b) and 3(c)). For proinflammatory cytokines, the level of TNF-*α* was comparable, while IL-6 was about 2-fold higher in B6/lpr mice. Production of IL-17, which is necessary for amplification of neutrophil recruitment, was also elevated in the lupus context (Figures 3(d)–3(f)). Therefore, Hi infection induced enhanced production of inflammation-related cytokines in the lupus-prone mice.

### 3.3. More Neutrophils Were Recruited to the Lupus Lung after Infection

**Figure 4:**
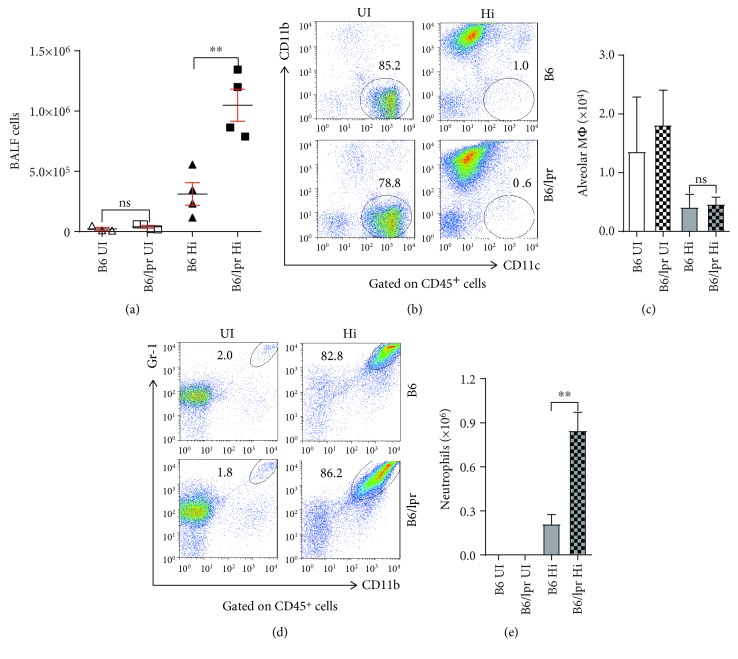
Tremendous neutrophils were recruited to the lupus lung after infection. B6 and B6/lpr mice were infected with Hi (B6 Hi and B6/lpr Hi). Uninfected mice were set as controls (B6 UI and B6/lpr UI). Two days after infection, all the mice were sacrificed and BALF cells were isolated for analyses. (a) Absolute numbers of BALF cells. (b, c) Alveolar macrophages (CD11b^−^CD11c^+^) and (d, e) neutrophils (CD11b^+^Gr-1^+^) were determined by surface staining. Data were expressed as means ± SEM. *n* = 3‐4 mice for each treatment group; ^∗∗^*p* < 0.01. ns: no significant difference. This experiment is representative of three individual experiments.

Alveolar macrophages and neutrophils are the first-line defence against invading pathogens. Therefore, we analysed them in the BALF. In the uninfected mice, the absolute numbers of BALF cells were comparable between groups (Figure 4(a)) and most of the cells were alveolar macrophages (CD11b^−^CD11c^+^) (Figures 4(b) and 4(c)). When infected by Hi, influxes of cells were observed in both groups (Figure 4(a)) and more than 80% of the recruited cells were neutrophils (CD11b^+^Gr-1^+^) (Figure 4(d)). Consistent with the elevated production of neutrophil chemoattractant detected in the lung, about 4-fold more neutrophils infiltrated into the lungs of B6/lpr mice (Figure 4(e)). Our data suggested that Hi infection induced augmented infiltration of neutrophils in B6/lpr mice.

### 3.4. Bacteria-Specific *γδ* T Cells from Lupus-Prone Mice Produced More IL-17

**Figure 5:**
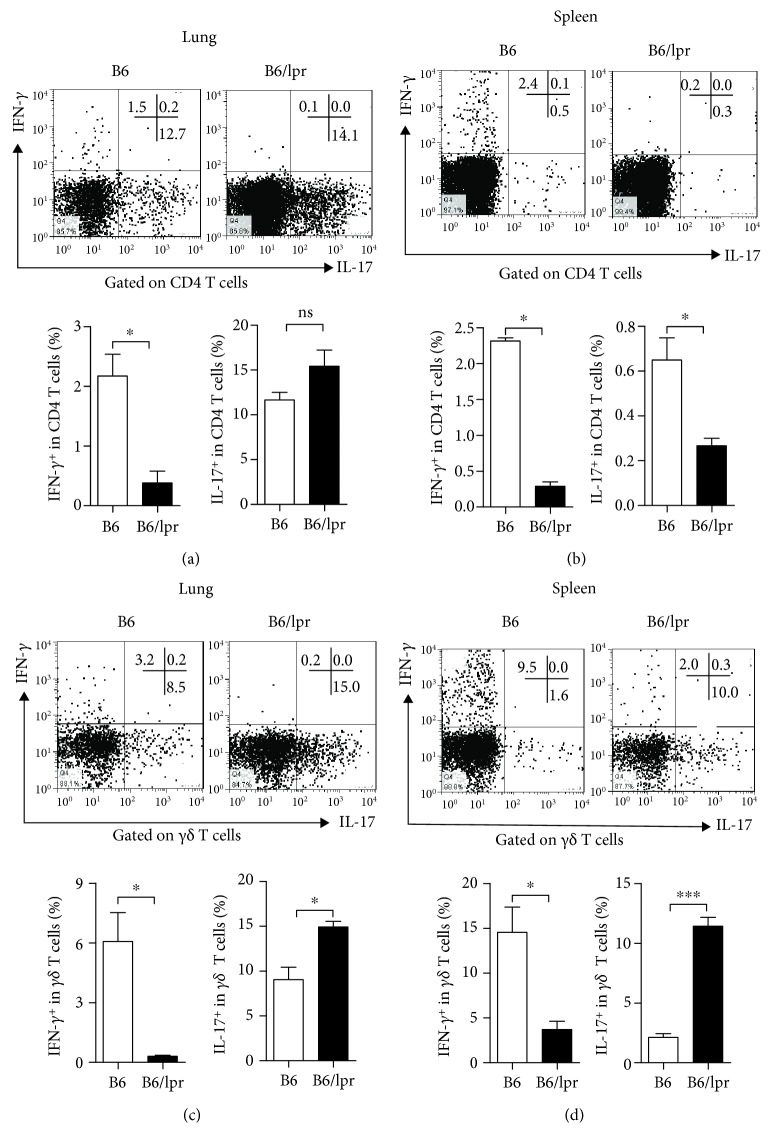
Bacteria-specific T cell responses in lupus-prone mice. Ten days after Hi infection, B6 and B6/lpr mice were sacrificed. Lung and spleen cells were isolated and stimulated with heat-killed Hi. Cytokine productions by CD4 and *γδ* T cells were determined by intracellular staining. (a, b) IFN-*γ* and IL-17 produced by CD4 T cells from the lung and spleen. (c, d) Percentages of IFN-*γ* and IL-17 secreting *γδ* T cells from the lung and spleen. Data were expressed as means ± SEM. *n* = 3‐4 mice for each treatment group. ^∗^*p* < 0.05; ^∗∗∗^*p* < 0.001. ns: no significant difference. This experiment is representative of three individual experiments.

Pathogen-specific T cells are critical for containment and elimination of infection [12]. In a recent study, we demonstrated that bacteria-specific Th1 (IFN-*γ* producing CD4 T cells) and Th17 (IL-17 producing CD4 T cells) cells played pivotal roles in restraining Hi infection [12]; thus, we first examined Th1 and Th17 responses induced by Hi in the lupus-prone mice. On Day 10 after infection, when Hi-specific T cell response reached the maximum, lung cells and splenocytes were isolated and stimulated with heat-killed Hi. The frequencies of IFN-*γ* and IL-17 producing CD4 T cells were determined by intracellular staining. Our results showed that in the lung, Hi infection induced comparable Th17 responses in both of the groups. But fewer Hi-specific Th1 cells were detected in B6/lpr mice (Figure 5(a)). In the spleen, ~9-fold decrease of Hi-specific Th1 cells was observed (Figure 5(b)).

It has been proved that *γδ* T cells are indispensable for clearing mucosal infection [13]. We found that in the lungs of B6 mice, most of the responding *γδ* T cells secreted IL-17 (IFN‐*γ*^+^ vs.IL‐17^+^ = ~2%vs.~10%) (Figure 5(c)), whereas in the spleen they preferred to produce IFN-*γ* (IFN‐*γ*^+^ vs.IL − 17^+^ = ~15%vs.~5%) (Figure 5(d)). However, a quite different pattern was observed in the B6/lpr mice. The majority of the responding *γδ* T cells from both lungs (~15%) and spleens (~12%) produced IL-17. A significant decrease of Hi-specific IFN-*γ* producing *γδ* T cells was observed in both lungs and spleens (Figures 5(c) and 5(d)). Together, these data showed that Hi infection induced enhanced IL-17 responses in the lupus-prone mice.

### 3.5. Bacteria-Specific Antibodies Were Significantly Increased in Infected Lupus-Prone Mice

**Figure 6:**
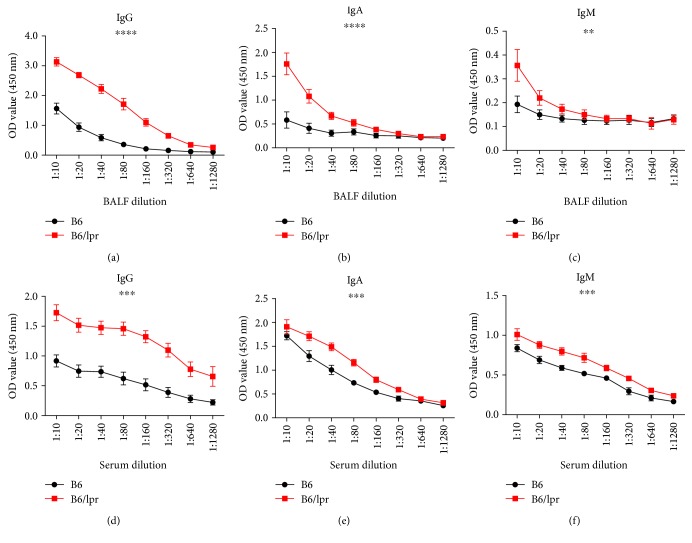
Bacteria-specific antibodies were significantly increased in the infected lupus-prone mice. Ten days after infection, B6 and B6/lpr mice were sacrificed. BALF and sera were collected. Hi-specific IgG (a, d), IgA (b, e), and IgM (c, f) were determined by ELISA. Data were expressed as means ± SEM and analysed by one-way ANOVA. *n* = 3 mice for each treatment group. ^∗∗^*p* < 0.01, ^∗∗∗^*p* < 0.001, and ^∗∗∗∗^*p* < 0.0001.

We next examined the other arm of the host's adaptive immune response, humoral immunity. We measured the levels of Hi-specific IgG, IgA, and IgM antibodies in the BALF and serum collected on Day 10 after infection. The concentrations of all the antibodies, especially IgG, in the infection site (BALF) were significantly higher in the B6/lpr mice (Figures 6(a)–6(c)). However, in the peripheral (serum), only the production of IgG was strongly elevated (Figure 6(d)), while IgA and IgM were slightly increased in the B6/lpr mice (Figures 6(e) and 6(f)). Together, Hi-specific humoral immunity was augmented in the context of SLE.

## 4. Discussion

Infection is one of the leading causes of morbidity and mortality in SLE patients. It has been well-accepted that the dysregulated immune system of SLE predisposes patients to infection. Correspondingly, infection is also believed to contribute to the progression of SLE. Thus, investigating infection-induced immune responses in the context of lupus is important for understanding the increased infection rate and helps to develop novel therapeutic approaches to prevent infection. To circumvent the confounding interferences by immunosuppressive drugs, lupus-prone mice were employed. Here, we chose the MRL/lpr strain, which has a spontaneous *FAS* mutation, and recapitulates many features of lupus [14]. We demonstrated that lupus-prone mice were capable to control the infection caused by opportunistic pathogen *H. influenzae*, but infection induced severer inflammation in the lung. During the infection, accumulated apoptotic cells were detected in the lupus lung. Notably, Hi infection induced augmented *γδ* T17 response and humoral immunity in the lupus-prone mice. These findings suggest that lupus context promotes the overactivation of the host's antibacterial immune response and causes detrimental effects.

Neutrophils act as the first-line defence against invaded bacteria. We found that B6/lpr mice had enhanced accumulation of neutrophils, but the elimination of infection did not speed up accordingly. This could be attributed to the intrinsic functional defects in the lupus neutrophils. It has been proved that reduced expression of Mac-1 contributed to the impaired phagocytosis of SLE neutrophils [15]. In an elegant study by Gresham et al., they demonstrated that due to the spontaneous production of TGF-*β*1, neutrophils from MRL/lpr mice exhibited a marked defect in the amplification of FcR-mediated phagocytosis. The inefficiency of neutrophils led to the impaired defence against both gram-negative and gram-positive bacterial infections in MRL/lpr mice [16, 17].

After elimination of bacteria, downregulation of the inflammatory response is crucial for avoiding tissue damage. Most of the infection-recruited inflammatory cells, such as neutrophils, will undergo apoptosis and be scavenged by phagocytes. Unfortunately, impaired macrophage phagocytic clearance of apoptotic neutrophils has been described in SLE patients [18]. Consistent with this study, we indeed observed an increased accumulation of apoptotic cells in the lupus lung after infection. According to our published data, reduced expression of CD206 by B6/lpr macrophages partly explained the deficient phagocytosis [19]. According to the previous study, inefficient clearance of apoptotic cells may subsequently lead to overwhelming inflammation and excessive lung injury [20]. Moreover, the apoptotic host cells would also enable the presentation of self-antigens and generate autoreactive Th17 cells, which in turn promote autoinflammation and autoantibody generation [21]. Thus, our data suggest that Hi infection itself may not lead to the severe illness of lupus-prone mice, but the incapability to remove apoptotic inflammatory cells may aggravate inflammation and promote SLE progression.

By attracting neutrophils, IL-17 plays a critical role in the clearance of infection [22]; however, overproduction of this cytokine contributes to the pathogenesis of several autoimmune diseases. CD4 T cells at first were believed to be the primary source of IL-17; however, it has been subsequently proved that *γδ* T cells are a more potent source of IL-17 [23]. Recently, *γδ* T17 cells have been proved to play an important role in regulating humoral immunity via promoting the differentiation of T follicular helper (Tfh) cells. Total immunoglobulins including IgA, IgG, and IgM in the serum and BALF were significantly decreased in *γδ* TCR^−/−^ mice [24]. In the present study, we found that Hi infection induced comparable antigen-specific Th17 but augmented *γδ* T17 responses in both lungs and spleens of B6/lpr mice. In parallel, an enhanced production of Hi-specific IgA, IgG, and IgM antibodies was also detected in the B6/lpr mice, implying that elevated *γδ* T17 responses may be responsible for the upregulation of the humoral immunity. According to the “bystander activation” theory, which describes a nonspecific activation of autoimmune cells caused by the inflammatory environment present during infection, it is reasonable to speculate that in the context of active SLE, the overactivated *γδ* T17 responses induced by infection might promote the production of autoantibodies and accelerate the progress of the disease. In this regard, investigating the relationship between the infection-induced *γδ* T17 response and the production of autoantibodies will provide new insight to elucidate the underlying mechanism of how infection initiates the SLE flares.

Another important finding of our study is the significantly decreased frequencies of Hi-specific Th1 cells. A similar phenomenon was also reported by Lieberman and Tsokos. They infected the lupus-prone murine strains B6/lpr and BXSB with the intracellular parasite *Toxoplasma gondii* and found that mice from both strains succumbed to infection acutely. The increased susceptibility was due to the decreased IFN-*γ* production by responding T cells [6]. Consistent with these experimental data, patients with SLE were also found to be susceptible to infection with Th1 pathogens such as *Salmonella* spp. [25]. This is interesting, because lupus pathogenesis has been attributed to the overproduction of IFN-*γ* and closely linked to the enhanced Th1 responses, particularly during late stages of disease [26]. Thus, there might be a difference in generating “protective” (pathogen-specific) and “pathogenic” (autoreactive) Th1 responses in the lupus-prone mice. Figuring out the underlying mechanisms will undoubtedly expand our understanding on the pathogenesis of SLE and help to develop new ways to handle the infection in SLE patients.

## 5. Conclusions

Our study provides a fresh perspective on the mechanisms causing increased susceptibility to infection in patients with autoimmune diseases. By discovering the enhanced *γδ* T17 and humoral responses induced by Hi infection, our study also helps to illustrate how infection initiates SLE flares. At the translational level, our data suggest that in addition to antibiotics, the correction of dysregulated T cell function in SLE patients should produce more favorable clinical outcomes following infections.

## Figures and Tables

**Table 1:** 

Parameter	Score per field		
	0	1	2
*A*: neutrophils in the alveolar space	None	1-5	>5
*B*: neutrophils in the interstitial space	None	1-5	>5
*C*: hyaline membrane formation	None	1	>1
*D*: proteinaceous debris filling the airspaces	None	1	>1
*E*: alveolar septal thickening	<2x	2x-4x	>4x

## Data Availability

The data used to support the findings of this study are included within the article.

## References

[1] Cervera R., Khamashta M. A., Font J. (2003). Morbidity and mortality in systemic lupus erythematosus during a 10-year period: a comparison of early and late manifestations in a cohort of 1,000 patients. *Medicine*.

[2] Petri M. (1998). Infection in systemic lupus erythematosus. *Rheumatic Disease Clinics of North America*.

[3] Ren Y., Tang J., Mok M. Y., Chan A. W. K., Wu A., Lau C. S. (2003). Increased apoptotic neutrophils and macrophages and impaired macrophage phagocytic clearance of apoptotic neutrophils in systemic lupus erythematosus. *Arthritis and Rheumatism*.

[4] Perez H. D., Hooper C., Volanakis J., Ueda A. (1987). Specific inhibitor of complement (C5)-derived chemotactic activity in systemic lupus erythematosus related antigenically to the Bb fragment of human factor B. *Journal of Immunology*.

[5] Kis-Toth K., Comte D., Karampetsou M. P. (2016). Selective loss of signaling lymphocytic activation molecule family member 4-positive Cd8+ T cells contributes to the decreased cytotoxic cell activity in systemic lupus erythematosus. *Arthritis & rheumatology*.

[6] Lieberman L. A., Tsokos G. C. (2014). Lupus-prone mice fail to raise antigen-specific T cell responses to intracellular infection. *PloS one*.

[7] Maddur M. S., Vani J., Lacroix-Desmazes S., Kaveri S., Bayry J. (2010). Autoimmunity as a predisposition for infectious diseases. *PLoS Pathogens*.

[8] Garcia-Guevara G., Rios-Corzo R., Diaz-Mora A. (2018). Pneumonia in patients with systemic lupus erythematosus: epidemiology, microbiology and outcomes. *Lupus*.

[9] Matute-Bello G., Downey G., Moore B. B. (2011). An official American Thoracic Society workshop report: features and measurements of experimental acute lung injury in animals. *American Journal of Respiratory Cell and Molecular Biology*.

[10] Antunes M. A., Abreu S. C., Cruz F. F. (2014). Effects of different mesenchymal stromal cell sources and delivery routes in experimental emphysema. *Respiratory Research*.

[11] Bordon J., Aliberti S., Fernandez-Botran R. (2013). Understanding the roles of cytokines and neutrophil activity and neutrophil apoptosis in the protective versus deleterious inflammatory response in pneumonia. *International Journal of Infectious Diseases*.

[12] Li W., Zhang X., Yang Y. (2018). Recognition of conserved antigens by Th17 cells provides broad protection against pulmonary *Haemophilus influenzae* infection. *Proceedings of the National Academy of Sciences of the United States of America*.

[13] Nielsen M. M., Witherden D. A., Havran W. L. (2017). *γδ* T cells in homeostasis and host defence of epithelial barrier tissues. *Nature Reviews Immunology*.

[14] Celhar T., Fairhurst A. M. (2016). Modelling clinical systemic lupus erythematosus: similarities, differences and success stories. *Rheumatology*.

[15] Zhou Y., Wu J., Kucik D. F. (2013). Multiple Lupus‐Associated ITGAM Variants Alter Mac‐1 Functions on Neutrophils. *Arthritis and Rheumatism*.

[16] Gresham H. D., Ray C. J., O'Sullivan F. X. (1991). Defective neutrophil function in the autoimmune mouse strain Mrl/Lpr. Potential role of transforming growth factor-beta. *Journal of Immunology*.

[17] Lowrance J. H., O'Sullivan F. X., Caver T. E., Waegell W., Gresham H. D. (1994). Spontaneous elaboration of transforming growth factor beta suppresses host defense against bacterial infection in autoimmune Mrl/Lpr mice. *The Journal of Experimental Medicine*.

[18] Munoz L. E., Lauber K., Schiller M., Manfredi A. A., Herrmann M. (2010). The role of defective clearance of apoptotic cells in systemic autoimmunity. *Nature reviews. Rheumatology*.

[19] Deng W., Chen W., Zhang Z. (2015). Mesenchymal stem cells promote Cd206 expression and phagocytic activity of macrophages through Il-6 in systemic lupus erythematosus. *Clinical Immunology*.

[20] Craig A., Mai J., Cai S., Jeyaseelan S. (2009). Neutrophil recruitment to the lungs during bacterial pneumonia. *Infection and Immunity*.

[21] Campisi L., Barbet G., Ding Y., Esplugues E., Flavell R. A., Blander J. M. (2016). Apoptosis in response to microbial infection induces autoreactive Th17 Cells. *Nature Immunology*.

[22] Iwanaga N., Kolls J. K. (2019). Updates on T helper type 17 immunity in respiratory disease. *Immunology*.

[23] Korn T., Petermann F. (2012). Development and function of interleukin 17–producing *γδ* T cells. *Annals of the New York Academy of Sciences*.

[24] Rezende R. M., Lanser A. J., Rubino S. (2018). *γδ* T cells control humoral immune response by inducing T follicular helper cell differentiation. *Nature Communications*.

[25] Iliopoulos A., Tsokos G. (1996). Immunopathogenesis and spectrum of infections in systemiclupus erythematosus. *Seminars in Arthritis and Rheumatism*.

[26] Ahn S. S., Park E. S., Shim J. S. (2017). Decreased ex vivo production of interferon-gamma is associated with severity and poor prognosis in patients with lupus. *Arthritis Research & Therapy*.

